# Healthy Eating Index-2020 and post-stroke depression (PSD): cross-sectional analysis of NHANES data

**DOI:** 10.1186/s12888-026-08402-5

**Published:** 2026-07-20

**Authors:** Can Xing, Zhenhui Lu, Xiaoming Guo, Xiangyang Zhu

**Affiliations:** Department of Neurology, Nantong First People’s Hospital, Nantong First People’s Hospital Affiliated to Southeast University, No. 666 Shengli Road, Nantong, Jiangsu 226000 China

**Keywords:** Healthy Eating Index-2020, Post-stroke depression, National Health and Nutrition Examination Survey, Dietary index for gut microbiota

## Abstract

**Background:**

Post-stroke depression (PSD) is a common complication after stroke and contributes to poor functional recovery and reduced quality of life. Dietary quality has been associated with depression in the general population, but evidence in individuals with stroke remains limited. This study examined the cross-sectional association between the Healthy Eating Index-2020 (HEI-2020) and PSD.

**Methods:**

This study included 962 adults with a history of stroke from seven cycles of the National Health and Nutrition Examination Survey from 2005 to 2018. Depressive symptoms were assessed using the Patient Health Questionnaire-9, with a score ≥ 10 indicating probable depression. Dietary quality was evaluated using HEI-2020 and categorized into tertiles. Multivariate logistic regression, restricted cubic spline analysis, and mediation analysis were used to assess associations and the statistical indirect association of the Dietary Index for Gut Microbiota (DI-GM).

**Results:**

After multivariate adjustment, each one-point increase in HEI-2020 score was associated with 2% lower odds of probable depression (PHQ-9-defined) (OR = 0.98). Compared with the low dietary quality group, the high dietary quality group had 59% lower odds (OR = 0.41). The dose–response relationship was nonlinear, with a significant inverse association observed only when HEI-2020 scores exceeded 57. This exploratory threshold may serve as a reference for future prospective studies but requires validation before clinical application. Furthermore, a significant interaction with race or ethnicity was identified (P for interaction = 0.033). DI-GM showed a significant indirect association (*P* = 0.04), but the direct and indirect components were in opposite directions, indicating an inconsistent mediation pattern. Given that DI-GM is a dietary proxy, these findings reflect statistical associations with a dietary pattern, not biological mediation. Future studies with direct microbiome measurements are needed to verify gut–brain axis involvement in PSD.

**Conclusions:**

In this cross-sectional study, HEI-2020 was inversely associated with PSD, with nonlinear and race-specific patterns. DI-GM showed a significant but inconsistent indirect association, suggesting a suppressive rather than mediating role. Findings are associational, not causal.

## Background

Stroke ranks as the second most common cause of death worldwide. High incidence and persistent disability after stroke have established it as a leading cause of long-term functional limitation [1, 2]. Accumulating evidence shows that stroke results in multidimensional impairments, including cognitive deficits, motor dysfunction, and psychiatric disorders [3]. Post-stroke depression (PSD) represents the most frequent and burdensome psychiatric complication, contributing substantially to overall disease burden among neuropsychiatric sequelae [3]. Meta-analyses report a cross-sectional prevalence ranging from 18% to 33% [4–7]. PSD further worsens motor and cognitive outcomes, reduces quality of life, and impairs recovery of activities of daily living and social reintegration [5, 8, 9]. Current understanding of the pathogenesis, risk factors, and management of PSD remains limited, highlighting the need for further investigation.

The pathophysiological mechanisms underlying PSD are complex and incompletely understood. This heterogeneous disorder does not arise from a single pathogenic pathway; instead, multiple interacting processes drive its onset and progression. Clarifying these mechanisms remains essential for developing targeted therapeutic strategies. Proposed pathways include monoaminergic neurotransmission dysfunction, reduced neurotrophic support, dysregulation of inflammatory hypothalamic–pituitary–adrenal axis activity, and glutamate-mediated excitotoxicity, among others [9]. Importantly, PSD differs from depression in the general population owing to the presence of acute brain injury, post-stroke neuroinflammation, and altered gut-brain axis signaling, as well as the frequent use of polypharmacy (e.g., antiplatelets, anticoagulants, and proton pump inhibitors), all of which may modify the relationship between diet and mood. These unique features suggest that findings from general depression studies cannot be directly extrapolated to PSD, underscoring the need for population-specific research.

Epidemiological evidence consistently links dietary nutrient intake to depression risk, with particular attention to micronutrients such as zinc, omega-3 polyunsaturated fatty acids, and vitamin D [10–12]. Certain dietary patterns, including the Dietary Approaches to Stop Hypertension diet, are also associated with a lower likelihood of depression [13–15]. Recent research in nutritional epidemiology has shifted from isolated nutrients to overall dietary patterns. These patterns reflect habitual combinations and frequencies of food and beverage intake over time and better capture real-world eating behaviors and their integrated effects on health outcomes than single-nutrient analyses.

The Healthy Eating Index (HEI), developed by the United States Department of Agriculture in accordance with the Dietary Guidelines for Americans, provides a measure of overall dietary quality by quantifying how closely the dietary pattern of an individual or population aligns with guideline recommendations. The index undergoes revision every five years to reflect updates in the dietary guidelines. Researchers have widely applied the HEI in dietary quality surveillance, studies examining associations between dietary patterns and health outcomes, evaluation of nutritional interventions, and analyses of food environments and policies [16].

HEI-2020 retains the same scoring framework as HEI-2015 [17] and has gained broad use in nutritional epidemiology. Higher HEI-2020 scores are associated with lower risks of cardiovascular disease, diabetes mellitus, and all-cause mortality [18, 19]. Wang et al. reported that lower HEI-2015 scores correlated with greater depressive symptom burden among adults in the United States [20], supporting a potential role of overall dietary quality in the development of depression. However, evidence on this association in PSD remains lacking. The present study aims to bridge this gap by examining the relationship between dietary quality, assessed using HEI-2020, and PSD. Addressing this gap has important clinical and translational implications: dietary quality is a modifiable factor that may be integrated into post-stroke rehabilitation programs. Exploring whether a threshold or dose-response relationship exists could provide a reference for the design of future prospective studies and may suggest dietary factors worthy of further investigation.

## Methods

### Study population

This cross-sectional study used data from the National Health and Nutrition Examination Survey (NHANES), an ongoing nationwide program conducted by the Centers for Disease Control and Prevention to assess the health and nutritional status of the non-institutionalized population in the United States. The survey employs a complex, multistage probability sampling design. The Ethics Review Board of the National Center for Health Statistics approved the protocol, and all participants provided written informed consent. All procedures adhered to the principles of the Declaration of Helsinki. This secondary analysis used publicly available data and did not require additional ethical approval.

**Fig. 1:**
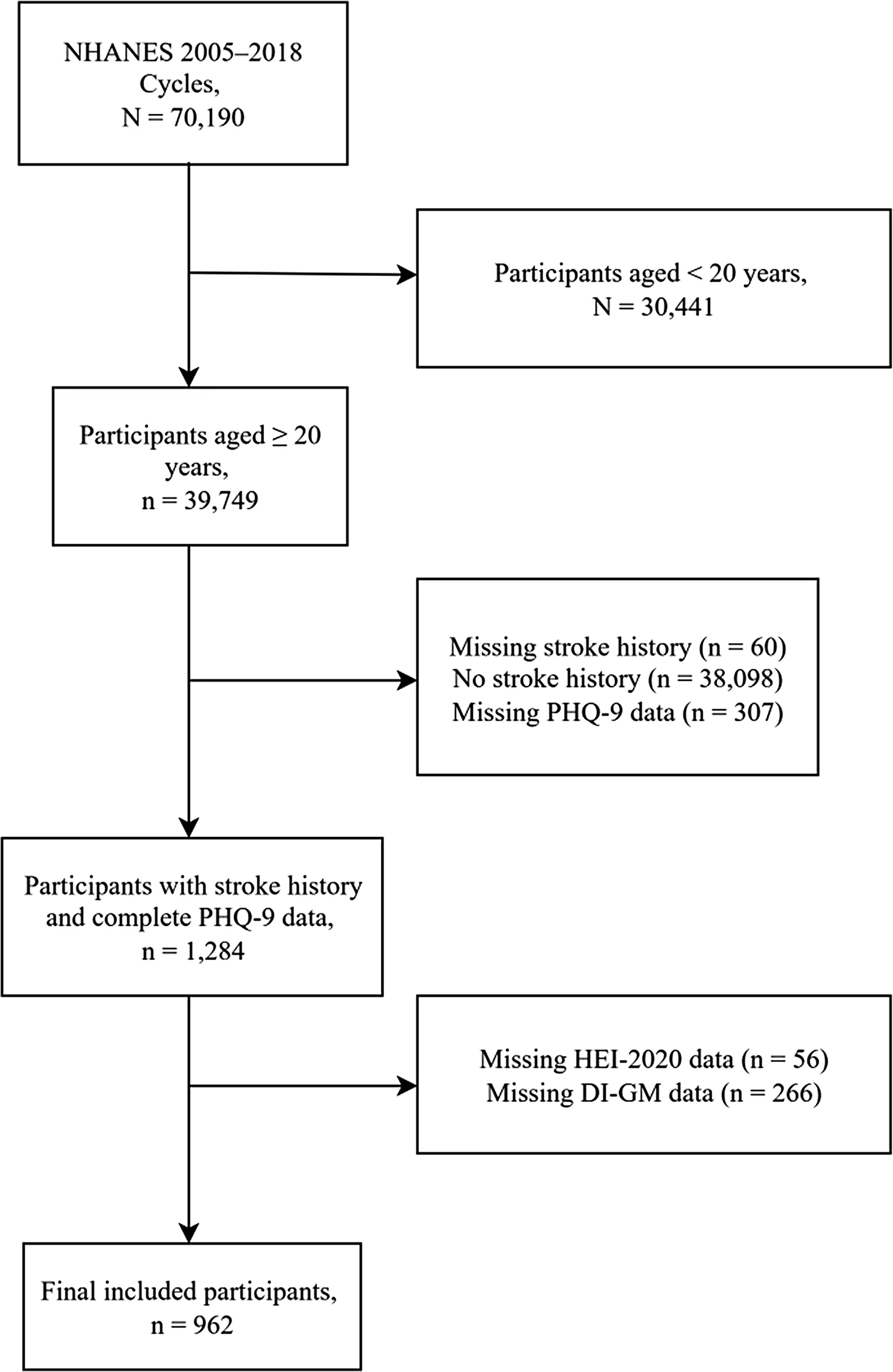
Flowchart of study population selection. Abbreviations: NHANES, National Health and Nutrition Examination Survey; PHQ-9, Patient Health Questionnaire-9 items

Data from seven consecutive NHANES cycles conducted between 2005 and 2018 were analyzed, including the 2005–2006, 2007–2008, 2009–2010, 2011–2012, 2013–2014, 2015–2016, and 2017–2018 cycles. The initial sample included 70,190 individuals. Inclusion criteria required age ≥ 20 years and availability of complete stroke history data. Stroke history was determined based on participants’ self-reported response to the question: ‘Has a doctor or other health professional ever told you that you had a stroke?‘. The NHANES dataset does not provide information on the timing of stroke onset or on pre-stroke depression history. Exclusion criteria included age < 20 years (n = 30,441), incomplete stroke history data (n = 60), absence of prior stroke (n = 38,098), missing Patient Health Questionnaire-9 (PHQ-9) scores (n = 307), unavailable HEI-2020 data (n = 56), and unavailable Dietary Index for Gut Microbiota (DI-GM) data (n = 266). The final analytic sample included 962 individuals. Figure 1 illustrates the participant selection process.

The covariates included sociodemographic characteristics, lifestyle factors, and comorbidities. Sociodemographic variables included age, sex, race or ethnicity, educational attainment, and poverty income ratio (PIR). Race or ethnicity was categorized as Mexican American, non-Hispanic White, non-Hispanic Black, or other. Educational attainment was grouped as less than high school, high school or equivalent, and college or above. PIR was analyzed as a continuous variable. Lifestyle factors included cigarette smoking and alcohol consumption. Smoking status classified individuals who reported smoking fewer than 100 cigarettes over a lifetime as non-smokers. Alcohol consumption was defined as intake of at least 12 alcoholic drinks during the 12 months preceding the survey.

Comorbidities included hypertension, diabetes mellitus, and cardiovascular disease. Hypertension was defined as a mean systolic blood pressure ≥ 140 mmHg or a mean diastolic blood pressure ≥ 90 mmHg based on three consecutive measurements, or use of antihypertensive medication. Diabetes mellitus was defined as glycated hemoglobin ≥ 6.5%, fasting plasma glucose ≥ 126 mg/dL, or use of glucose-lowering medication or insulin. Cardiovascular disease was identified based on self-reported questionnaire data.

### Assessment of depression using the PHQ-9 scale

Depressive symptoms were assessed using the PHQ-9, a self-report instrument based on the diagnostic criteria for depression in the Diagnostic and Statistical Manual of Mental Disorders, Fourth Edition. The PHQ-9 includes nine items, each scored from 0 to 3, yielding a total score ranging from 0 to 27, with higher scores indicating greater symptom severity. The instrument demonstrates strong screening performance, with sensitivity and specificity of approximately 88% [21]. In accordance with prior literature, a PHQ-9 score ≥ 10 defined clinically relevant depressive symptoms, a threshold that provides an optimal balance between sensitivity and specificity for identifying depression [22].

In this study, we employed the term “post-stroke depression” (PSD), referring to the depressive symptoms (PHQ-9 ≥ 10) identified among participants who reported a history of stroke in their self-reports. This operational definition is consistent with previous studies based on NHANES. However, since NHANES did not provide data on the time of stroke occurrence or pre-stroke depression, our research results reflect a cross-sectional association among stroke survivors, rather than a strictly temporally defined post-stroke depression.

### Healthy Eating Index-2020

Dietary quality was evaluated using HEI-2020, a standardized measure developed by the United States Department of Agriculture and the National Cancer Institute in alignment with the 2020–2025 Dietary Guidelines for Americans. The index yields a total score ranging from 0 to 100, with higher scores indicating better dietary quality [17].

Dietary intake data were obtained from two consecutive 24-hour dietary recalls conducted within NHANES, including one weekday and one weekend day. A multi-pass recall approach was employed to reduce recall bias, and the mean of the two assessments was used for analysis. HEI-2020 includes 13 components, comprising nine adequacy components and four moderation components. All components were scored using an energy density approach per 1000 kcal and calculated according to the standardized protocol of the United States Department of Agriculture.

Participants with less than 80% data completeness were excluded, whereas those with 80% to 99% completeness underwent imputation for missing values.

### Dietary index for gut microbiota

DI-GM is a standardized dietary score derived from evidence linking specific food components to adult gut microbiota composition. Preliminary validation has been performed using associations with microbial diversity biomarkers within the NHANES dataset. The index integrates multiple dietary components into a composite score ranging from 0 to 14, with higher scores indicating a stronger promotive effect on gut microbial diversity and closer alignment with a healthy gut microecosystem [23].

The index includes 10 protective components and four detrimental components. Protective components include fermented dairy products, chickpeas, soybeans, whole grains, dietary fiber, cranberries, avocados, broccoli, coffee, and green tea. Detrimental components include refined grains, red meat, processed meats, and dietary patterns in which fat intake exceeds 40% of total energy intake.

Scoring was based on sex-specific median intake levels. Each protective component received one point when intake exceeded the median and 0 points otherwise. Each detrimental component received one point when intake fell below the median and 0 points otherwise. The total DI-GM score was calculated as the sum of all component scores, yielding a final score between 0 and 14 [23].

### Data analysis and statistical methods

Data management and statistical analyses were conducted using Zstats (www.zstats.net) and R software version 4.4.0 (April 24, 2024). Continuous variables are presented as means with standard errors, and categorical variables as frequencies and percentages (n, %). Group comparisons were performed using the chi-square test for categorical variables and the independent samples t-test for continuous variables.

In the classification analysis, the participants were divided into tertiles based on the distribution of the HEI-2020 score in the study population: the low dietary quality group (≤ 48.25), the moderate dietary quality group (48.25–57.27), and the high dietary quality group (> 57.27). These cut-off points correspond to the 33rd and 66th percentiles of the HEI-2020 distribution in this study. HEI-2020 was analyzed both as a continuous variable (with each additional point) and as a categorical variable using the above tertiles. Tertile grouping was selected to facilitate a simple and intuitive comparison of low, moderate, and high dietary quality, while maintaining sufficient statistical power and model stability.

Three logistic regression models assessed the association between HEI-2020 and PSD. Model 1 was unadjusted. Model 2 adjusted for age, sex, and race or ethnicity. Model 3 further adjusted for educational level, smoking status, alcohol consumption, hypertension, diabetes mellitus, coronary heart disease, PIR, average 2-day energy intake, and metabolic equivalent of task (MET). In the analysis sample, the number of PSD events (PHQ-9 ≥ 10) was 182 cases. The fully adjusted model (Model 3) contained 15 parameters, and the event ratio (EPV) was approximately 12.1, which exceeded the minimum standard of at least 10 events per variable recommended to avoid model overfitting. These covariates were selected a priori based on established risk factors for depression and known confounders of the diet–depression relationship, including sociodemographic status, lifestyle behaviors, comorbidities, and energy intake, to minimize potential confounding and isolate the independent association of dietary quality.

We conducted stratified subgroup analyses by sex, race/ethnicity, educational level, smoking status, alcohol consumption, hypertension, diabetes, and cardiovascular disease. Interaction terms were incorporated to test heterogeneity of associations across subgroups. Restricted cubic spline (RCS) regression was applied to characterize the nonlinear dose–response relationship between HEI-2020 and PSD. Knots were set at the 5th, 35th, 65th, and 95th percentiles of HEI-2020 scores, with the median value of 52.91 as the reference. Wald tests were used to assess the overall association and nonlinearity. All RCS models were adjusted for the full set of covariates defined in Model 3. Mediation analysis decomposed the total cross-sectional association between HEI-2020 and PSD into direct and indirect components via DI-GM. DI-GM was chosen as a statistical proxy rather than a direct measurement of gut microbiota composition, as this index was constructed based on dietary components reported to correlate with gut flora in prior literature. All mediation outputs serve purely as statistical descriptions and cannot support causal inference. Due to the cross-sectional design, temporal ordering of variables cannot be established; the terms “direct” and “indirect” only describe statistical components of the overall association and do not represent biological pathways. This analysis quantifies the proportion of the HEI-2020–PSD association statistically accounted for by the DI-GM dietary pattern.

All analyses used two-sided tests, with statistical significance defined as *P* < 0.05. All analyses incorporated the appropriate NHANES sampling weights, stratification, and clustering variables to account for the complex survey design and to generate nationally representative estimates.

## Results

In total, 962 participants were included, comprising 321 (33.37%) in the low HEI-2020 group, 321 (33.37%) in the moderate group, and 320 (33.26%) in the high group. Significant differences across groups were observed for age, body mass index, DI-GM, HEI-2020 score, educational level, smoking history, and depression status (*P* < 0.05). PIR, metabolic equivalent of task, average two-day energy intake, sex, race or ethnicity, hypertension, diabetes mellitus, coronary heart disease, and alcohol consumption did not differ significantly across groups (*P* > 0.05; Table 1).

**Table 1:** Baseline characteristics of the study population

Variable	Total (*n* = 962)	Low HEI-2020 Group (*n* = 321)	Moderate HEI-2020 Group (*n* = 321)	High HEI-2020 Group (*n* = 320)	*P*
Age, Mean (SE)	63.41 (0.83)	58.81 (1.07)	64.80 (1.07)	66.56 (1.72)	< 0.001
PIR, Mean (SE)	2.34 (0.08)	2.13 (0.12)	2.33 (0.15)	2.56 (0.14)	0.066
MET, Mean (SE)	2143.70 (287.98)	2855.43 (750.28)	1442.27 (301.83)	2152.32 (330.10)	0.110
Average two-day energy intake, Mean (SE)	1802.29 (36.23)	1740.01 (50.82)	1849.66 (72.53)	1815.88 (60.31)	0.407
DI-GM, Mean (SE)	4.87 (0.10)	3.79 (0.15)	4.85 (0.11)	5.96 (0.16)	< 0.001
HEI 2020, Mean (SE)	53.18 (0.47)	41.75 (0.37)	52.67 (0.20)	65.10 (0.58)	< 0.001
Sex, n(%)					0.443
Male	469 (42.52)	166 (46.75)	151 (40.94)	152 (39.93)	
Female	493 (57.48)	155 (53.25)	170 (59.06)	168 (60.07)	
Education, n (%)					0.001
Less than high school	301 (25.34)	106 (30.12)	103 (23.12)	92 (22.83)	
High school or equivalent	278 (32.26)	104 (38.45)	93 (34.43)	81 (23.87)	
College or above	383 (42.41)	111 (31.43)	125 (42.45)	147 (53.30)	
Race, n (%)					0.458
Mexican American	82 (5.27)	24 (5.08)	34 (6.72)	24 (3.96)	
Non-Hispanic White	497 (69.99)	161 (68.55)	170 (70.75)	166 (70.66)	
Non-Hispanic Black	262 (15.22)	98 (17.91)	81 (14.30)	83 (13.49)	
Other Race	121 (9.52)	38 (8.46)	36 (8.23)	47 (11.89)	
Hypertension, n (%)					0.983
No	171 (23.48)	55 (23.53)	63 (22.98)	53 (23.94)	
Yes	791 (76.52)	266 (76.47)	258 (77.02)	267 (76.06)	
Diabetes, n (%)					0.606
No	535 (59.55)	180 (60.41)	175 (56.44)	180 (61.88)	
Yes	427 (40.45)	141 (39.59)	146 (43.56)	140 (38.12)	
CVD, n (%)					0.166
No	782 (80.06)	266 (81.50)	261 (83.64)	255 (74.97)	
Yes	180 (19.94)	55 (18.50)	60 (16.36)	65 (25.03)	
Depression, n (%)					0.001
No	780 (80.61)	239 (73.64)	258 (78.40)	283 (89.83)	
Yes	182 (19.39)	82 (26.36)	63 (21.60)	37 (10.17)	
Smoking status, n (%)					0.026
No	383 (37.59)	122 (36.65)	124 (38.44)	137 (37.67)	
Yes	579 (62.41)	199 (63.35)	197 (61.56)	183 (62.33)	
Alcohol consumption status, n (%)					0.956
No	376 (37.45)	121 (36.75)	123 (38.57)	132 (37.00)	
Yes	570 (62.55)	194 (63.25)	194 (61.43)	182 (63.00)	

Abbreviations: SE, standard error; PIR, poverty-income ratio; MET, metabolic equivalent of task; DI-GM, Dietary Index for Gut Microbiota; HEI-2020, Healthy Eating Index-2020; CVD, cardiovascular disease

Multivariable logistic regression demonstrated an inverse association between HEI-2020 and PSD. After adjustment for demographic characteristics, lifestyle factors, and chronic disease history (Model 3), each one-point increase in HEI-2020 score was associated with a 2% lower odds of PSD (odds ratio [OR] = 0.98, 95% confidence interval [CI]: 0.96–0.99, *P* = 0.036; Table 2).

**Table 2:** Logistic regression analysis of the association between HEI-2020 and post-stroke depression

Variable	Model 1		Model 2		Model 3	
	OR (95%CI)	*P*	OR (95%CI)	*P*	OR (95%CI)	*P*
HEI 2020	0.97 (0.95–0.98)	< 0.001	0.98 (0.96–0.99)	0.005	0.98 (0.96–0.99)	0.036
HEI-2020 groups						
Low HEI-2020 group	1.00 (Reference)		1.00 (Reference)		1.00 (Reference)	
Moderate HEI-2020 group	0.77 (0.43–1.37)	0.377	0.94 (0.52–1.69)	0.827	0.92 (0.53–1.62)	0.782
High HEI-2020 group	0.32 (0.19–0.52)	< 0.001	0.39 (0.22–0.68)	0.001	0.41 (0.23–0.74)	0.004

**OR**: Odds ratio; **CI**: Confidence interval

Model 1 was unadjusted for any confounding factors

Model 2 was adjusted only for age, sex, and race

Model 3 was further adjusted for education level, alcohol consumption, smoking history, hypertension, diabetes, cardiovascular disease, poverty income ratio (PIR), metabolic equivalent of task (MET), and average two-day energy intake, based on Model 2

Tertile stratified analysis demonstrated that participants in the high HEI-2020 group had significantly lower odds of PSD relative to the low HEI-2020 group (OR = 0.41, 95% CI: 0.23–0.74, *P* = 0.004), corresponding to a 59% reduction in the odds of depressive symptoms after stroke. This association remained consistent after sequential adjustment, and the trend test supported a dose–response relationship (*P* < 0.05). The moderate HEI-2020 tertile exhibited a non-significant reduction in odds of PSD (OR = 0.92, 95% CI: 0.53–1.62, *P* = 0.782), consistent with a threshold effect: the inverse association was only apparent among participants with elevated HEI-2020 scores. Collectively, these results demonstrate that higher HEI-2020 values are independently linked to reduced odds of PSD. Subgroup analyses identified significant effect modification by race or ethnicity (P for interaction = 0.033). A significant association was observed only among non-Hispanic White individuals, whereas other racial and ethnic groups showed no significant association, suggesting possible population-specific differences in this relationship.

**Fig. 2:**
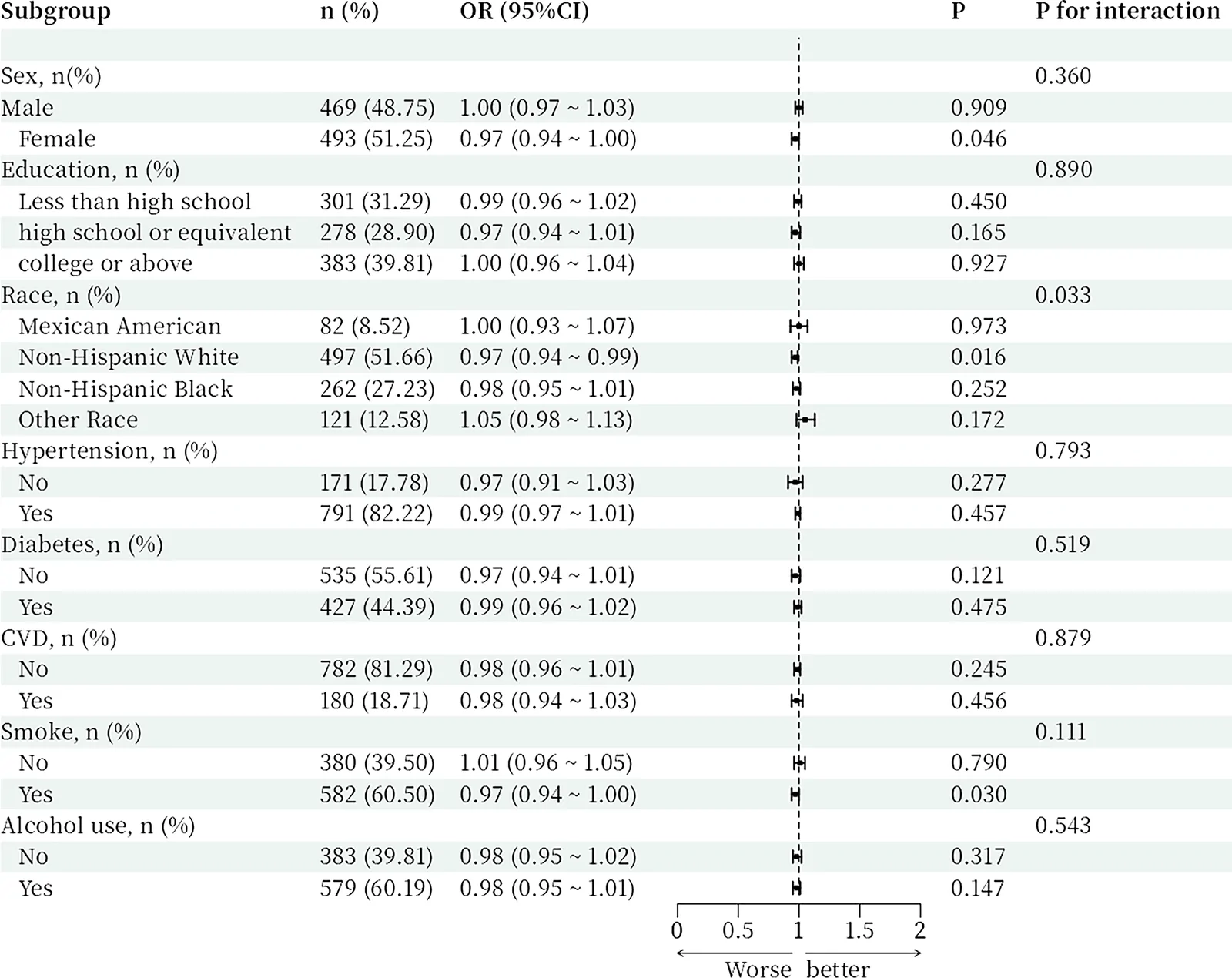
Stratified analysis of the association between hEI-2020 and post-stroke depression

Sex, educational level, hypertension, diabetes mellitus, coronary heart disease, and alcohol consumption did not significantly modify the association (P for interaction > 0.05). Smoking history did not show a statistically significant interaction (P for interaction = 0.111). However, stratified analysis demonstrated a significant association among individuals with a history of smoking, with no corresponding association among those without such a history. This pattern suggests a potential modifying effect of smoking status that warrants further evaluation in larger studies (Fig. 2).

**Fig. 3:**
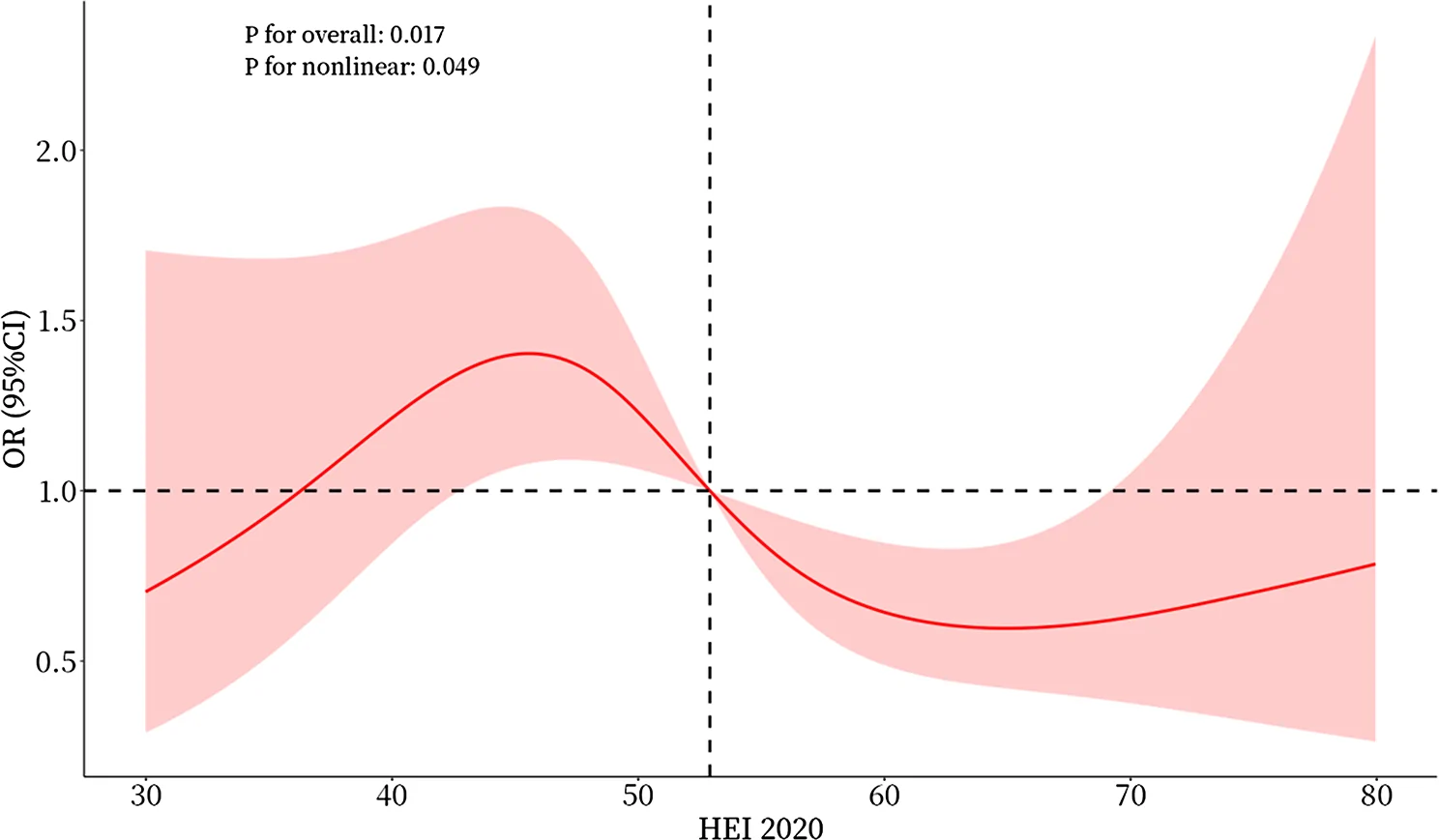
Restricted Cubic Spline (RCS) Curve of HEI-2020 and post-stroke depression. Note: The curve represents adjusted odds ratios (ORs), with the pink shaded area indicating the 95% confidence interval (CI). The vertical dashed line denotes the reference value (52.91); the horizontal dashed line indicates OR = 1. P for overall represents the P-value for overall association, and P for nonlinear represents the P-value for nonlinearity

A restricted cubic spline model assessed the dose–response relationship between HEI-2020 score and outcome odds, with knots placed at the 5th, 35th, 65th, and 95th percentiles of HEI-2020 (36.66, 48.75, 56.72, and 73.82, respectively), with 52.91 as the reference. The multivariable model adjusted for sex, educational level, smoking history, race or ethnicity, hypertension, diabetes mellitus, alcohol consumption, coronary heart disease, age, PIR, physical activity, energy intake, and body mass index (Fig. 3). The analysis demonstrated a significant nonlinear association (P for nonlinearity = 0.049; P for overall association = 0.017). Below the reference HEI value, the odds of PSD initially increased and then decreased as dietary quality improved. The highest likelihood of depressive symptoms appeared at HEI scores of 40–50 (OR range: 1.20–1.65), which reflected elevated risk among individuals with moderately suboptimal diets. Beyond this reference threshold, risk decreased gradually. Scores of 70 to 80 were linked to an approximately 15%–20% lower probability of PSD, in line with a potential inverse correlation between superior dietary quality and depressive symptoms (Fig. 3).

Consistent with NHANES’s cross-sectional design, all mediation outputs are treated as purely statistical correlations and cannot support causal biological inferences. We decomposed the overall association between HEI-2020 and PSD into direct and indirect components through DI-GM. The total association estimate was − 0.0686 (95% CI: −0.1123 to − 0.0100, *P* < 0.001), indicating that higher dietary quality was associated with lower odds of PSD. The direct component estimates were − 0.1033 (95% CI: −0.1650 to − 0.0300, *P* < 0.001) and − 0.1111 (95% CI: −0.1767 to − 0.0300, *P* < 0.001), with corresponding indirect components of 0.0425 (95% CI: 0.0097–0.0800, *P* = 0.04) and 0.0347 (95% CI: 0.0085–0.0600, *P* = 0.04). Both direct and indirect components reached statistical significance. This opposing-sign phenomenon is known as inconsistent mediation, or statistical suppression: the negative direct association is partially offset by the positive indirect component, thereby weakening the magnitude of the total association. This directional discrepancy does not indicate heterogeneity across analytical models, as all models adopted the same exposure-mediator-outcome framework. The proportion of the overall association statistically mediated through DI-GM was significant for both mediation estimates. Notably, DI-GM is only a surrogate dietary indicator without direct gut microbiota sequencing measurements. Coupled with the inherent limitation of cross-sectional data that cannot verify variable temporal ordering, these significant indirect statistical links do not constitute evidence of a biologically functional gut–brain mediation mechanism (Table 3).

**Table 3:** Mediation analysis of DI-GM in the HEI-2020–PSD association: Direct and Indirect Components

Effect Type	Estimate (95% Confidence Interval)	*P* value
Average Causal Mediation Effect (ACME)	0.039 (0.009, 0.07)	0.04
Average Direct Effect (ADE)	-0.107 (-0.171, -0.03)	< 0.001
Total Effect	-0.069 (-0.112, -0.01)	< 0.001
Proportion Mediated (%)	55.5	0.04

Note: Mediation analysis was performed using the quasi-Bayesian confidence interval method to statistically decompose the association between HEI-2020 and PSD into direct and indirect components. The terms used in this table (e.g., ‘component’) are statistical descriptors and do not imply causal relationships, given the cross-sectional design. The inconsistent pattern—opposing signs of the direct (negative) and indirect (positive) components—is consistent with a statistical suppression pattern, not classical mediation. This does not reflect heterogeneity across analyses; all models examined the same exposure-mediator-outcome relationship. *N* = 823, with 50 simulations

Sensitivity Analysis To assess the robustness of our findings within a causal inference framework, we performed a sensitivity analysis using a parsimonious adjustment set, which excluded potential mediators (hypertension, diabetes, and cardiovascular disease) to avoid overadjustment bias. The results remained highly consistent with the primary model. In the parsimonious model, each one-point increase in HEI-2020 was associated with an OR of 0.98 (95% CI: 0.97–0.99, *P* = 0.042) for PSD, and the high versus low HEI-2020 tertile showed an OR of 0.45 (95% CI: 0.26–0.78, *P* = 0.005). These estimates were materially unchanged from the primary model (OR = 0.98, *P* = 0.036 for continuous; OR = 0.41, *P* = 0.004 for high vs. low tertile), indicating that our findings are stable and unlikely to be biased by overadjustment for comorbid conditions.

## Discussion

Using data from NHANES 2005–2018, this study evaluated the cross-sectional association between HEI-2020 and depressive symptoms among stroke survivors (operationalized as PSD herein) and explored the statistical indirect effect of DI-GM within this relationship. Given NHANES’s cross-sectional design, all results should be interpreted as correlational rather than causal. We observed a robust, significant inverse association between HEI-2020 scores and PSD odds after adjusting for multiple confounders. Dose–response modelling revealed a nonlinear relationship, whereby the inverse association became more prominent at HEI-2020 values above 57.27 (the 66th percentile of our study sample). Subgroup analyses suggested effect modification by race/ethnicity. Mediation analysis further detected a statistically significant indirect pathway via DI-GM, yet the effect pattern represented inconsistent mediation: the direct component was negative, whereas the indirect component carried a positive estimate. Critically, DI-GM serves only as a dietary proxy rather than a direct biomarker of gut microbiota. Accordingly, this mediation result merely reflects a statistical correlation with a specific dietary pattern and cannot validate any biological gut–brain axis mechanism. Collectively, these cross-sectional observations show that overall dietary quality quantified by HEI-2020 correlates with depressive symptoms among individuals with self-reported stroke histories, highlighting diet as a promising target for future prospective cohorts to test potential causal links.

Each one-point increase in HEI-2020 was associated with 2% lower odds of PSD (OR = 0.98, 95% CI: 0.96–0.99). Relative to participants with low dietary quality, those with HEI-2020 scores above 57.27 had 59% lower odds of depressive symptoms after stroke (OR = 0.41, 95% CI: 0.23–0.74). The 57.27 cutoff corresponds to the 66th percentile within our study sample and should not be treated as a clinically validated threshold. Although this exploratory cutoff may serve as a reference for future prospective studies, external independent validation is required before any clinical application can be considered. These findings align with prior evidence linking overall dietary patterns to depression. Wang et al., using NHANES data, reported that lower HEI-2015 scores were associated with increased depressive symptoms among adults in the United States [20]. In addition, studies in stroke populations have shown that adherence to healthy dietary patterns, such as the Mediterranean diet, is associated with lower PSD odds [14, 24]. The present study extends this evidence to HEI-2020, the most recent dietary guideline-based index, and focuses on a high-risk post-stroke population, providing updated and population-specific data. Notably, the association between HEI-2020 and PSD followed a nonlinear pattern. The highest odds of PSD occurred among participants with HEI-2020 scores of 40–50 (OR range: 1.20–1.65). This reveals that individuals with moderately poor dietary quality exhibited a higher likelihood of depressive symptoms than those with extremely low-quality diets [25, 26]. This is a data-supported observation, but the explanation remains speculative.

Furthermore, this nonlinear trend may reflect the complex nutritional landscape following stroke. Extremely poor diet often accompanies general deterioration of physical health, while moderately suboptimal dietary quality may signal hidden malnutrition or other unmeasured health confounders. Without longitudinal data, all such interpretations remain speculative. Within our sample, PSD odds declined steadily when HEI-2020 scores exceeded approximately 57 points, suggesting a potential threshold effect. Nevertheless, the nonlinear test only yielded marginal statistical significance (*P* = 0.049), and this cutoff value is sample-specific. Thus this observation is purely exploratory and requires validation in independent cohorts. While these results can guide the design of future prospective research to verify potential causal links, no causal benefits can be concluded from this cross-sectional dataset. Beyond this dose–response pattern, subgroup stratification revealed a significant inverse HEI-2020–PSD association exclusively among non-Hispanic White participants (P for interaction = 0.033), with no meaningful correlation detected in other racial and ethnic subgroups. This result warrants cautious interpretation. Small sample sizes in non-White subgroups likely limited statistical power, making weak associations undetectable. Moreover, the HEI-2020 framework was built around American dietary guidelines, which better align with eating habits of non-Hispanic White populations and fail to capture protective nutritional elements embedded in the traditional diets of other ethnic groups [27]. Genetic, metabolic and sociocultural disparities may also drive such group differences, though these mechanisms remain unconfirmed [28–30]. As an exploratory subgroup outcome, this result is merely hypothesis-generating and calls for replication in larger, more racially diverse cohorts. Smoking status did not produce a significant interaction term (P for interaction = 0.111). Still, stratified analysis identified a stronger inverse association between higher HEI-2020 scores and lower depressive symptoms in participants with a smoking history versus never-smokers. Even though this stratified trend generates new research hypotheses, it must be interpreted cautiously given the non-significant overall interaction. Cigarette smoking elevates oxidative stress and systemic inflammation [31], which may amplify post-stroke neuroinflammation and thereby modulate the diet-depression relationship. Accordingly, higher dietary quality may carry a more pronounced inverse association with PSD among stroke survivors with prior smoking exposure.

When interpreting these cross-sectional correlations, reverse causation constitutes an important concern. The NHANES cross-sectional design cannot establish the temporal order between higher dietary quality and depressive symptoms. Clinically, post-stroke depressive presentations including apathy, fatigue and low motivation may suppress appetite and lower total food intake, while also creating barriers to meal planning and cooking—all of which contribute to reduced HEI-2020 scores. This reverse pathway is especially prominent among stroke survivors, as physical disability further complicates adherence to healthy dietary patterns [49, 50]. Thus, the observed associations may partly reflect an effect of depressive symptoms on diet, rather than the reverse [51]. Longitudinal prospective cohorts with repeated measurements of dietary habits and mood status are required to disentangle this temporal sequence and differentiate these two competing directional hypotheses [51].

The mediation analysis identified a statistically significant indirect association through DI-GM; however, the mediation pattern was inconsistent. Consistent with the cross-sectional design and the proxy nature of DI-GM, these mediation findings should be interpreted as statistical associations with a dietary pattern rather than as evidence of a causal biological pathway involving the gut microbiome. The direct component was negative, suggesting an inverse association with higher HEI-2020, whereas the indirect component through DI-GM was positive. Although higher dietary quality increased DI-GM scores (β = 1.124, P < 0.001), higher DI-GM scores were associated with increased odds of depression (OR = 1.235, P = 0.035), yielding a positive indirect component. These mediation results are statistically significant, but the unexpected direction of the indirect component warrants caution. This finding should not be interpreted as evidence that foods traditionally considered ‘gut-friendly’ are harmful in post-stroke populations. Instead, the inconsistent pattern may reflect the limitations of DI-GM as a dietary summary score in capturing post-stroke gut microbial dynamics, residual confounding, or reverse causation. While alterations in the gut–brain axis have been observed after stroke [32–40], we lack direct microbiological data to validate or refute any biological interpretation of these findings. Therefore, these mediation results should be viewed as exploratory and hypothesis-generating, not as evidence for or against gut microbiota-mediated pathways in PSD. If future studies confirm the inverse association between HEI-2020 and PSD, the underlying mechanisms, if causal, may not be driven by the pathways captured by DI-GM, but rather could involve other pathways such as anti-inflammatory, antioxidant, or neurotrophic ones. However, such mechanistic interpretations remain speculative without direct measurement of these pathways. Future studies incorporating direct assessment of microbiota composition, function, and metabolites are needed to test the biological plausibility of gut–brain axis involvement in PSD.

The pathogenesis of PSD is complex and incompletely understood. Current evidence supports a multifactorial model in which stroke-related biological changes and psychosocial factors interact through neurobiological pathways to promote depression [41, 42]. Key mechanisms include inflammatory responses, oxidative stress, and psychosocial influences. Post-stroke brain injury activates innate immune pathways and increases production of pro-inflammatory cytokines, including tumor necrosis factor, interleukin-6, and high-sensitivity C-reactive protein. These mediators contribute to neuronal injury and disrupt monoamine neurotransmitter synthesis and metabolism [43–45]. Oxidative stress also plays a central role. Excess reactive oxygen species generated during the acute phase of stroke induce lipid peroxidation, protein oxidation, and DNA damage, forming a molecular basis for the development of PSD [46]. In parallel, psychosocial factors, including persistent physical impairment, cognitive decline, and reduced social support, further increase susceptibility to depression after stroke [41]. Against this multifactorial pathogenic backdrop, our cross-sectional data indicate that dietary quality may interact with inflammatory and oxidative stress pathways, though causal inference remains unattainable [52, 53]. This exploratory set of results generates novel hypotheses. If replicated in longitudinal investigations, they could inform future efforts to develop optimized preventive and therapeutic strategies for PSD.

The findings of this study have several implications for clinical prevention and management of PSD. HEI-2020, aligned with the Dietary Guidelines for Americans, provides a practical tool for dietary assessment and risk stratification in stroke populations. The observed threshold of 57.27 was associated with 59% lower odds, offering a potential quantitative reference for hypothesis-generating research. The inconsistent mediation pattern observed in this cross-sectional analysis should be interpreted with caution. Given the proxy nature of DI-GM and the lack of direct microbiome measurements, this pattern does not provide evidence for or against the potential benefits of dietary patterns that promote gut health. Instead, it highlights the need for prospective studies with direct biological measurements to test whether diet–microbiota–depression pathways exist and whether they are clinically relevant. Effective management may require integrated approaches that combine attention to dietary quality with strategies addressing gut function, appropriate medication use, and psychosocial support [47]. The observed race-specific associations further indicate the importance of population heterogeneity in dietary research and intervention design. Tailored nutritional strategies that account for cultural dietary practices may improve effectiveness and help reduce disparities in mental health outcomes [48].

This study has several limitations. First, the cross-sectional NHANES design prevents causal inference. Mediation analysis only yielded statistical indirect effects rather than genuine causal pathways; prospective cohorts are required for validation. Reverse causation is also plausible: post-stroke depressive symptoms may reduce dietary intake and self-care, meaning the observed correlation could reflect depression altering diet instead of diet affecting mood, which requires longitudinal data to verify temporal ordering. Second, stroke history and depressive symptoms relied on self-report and screening tools, creating risks of recall bias and misclassification. We defined depressive symptoms via a PHQ-9 cutoff ≥ 10, a validated screening threshold with ~ 88% sensitivity, yet it cannot replace formal clinical depression diagnosis and may produce false positives. Non-differential misclassification would tend to attenuate associations toward the null, but future work should adopt standardized clinical assessments to enhance diagnostic accuracy. Third, DI-GM was calculated from one 24-hour dietary recall and cannot fully capture long-term dietary habits. Missing green tea intake data in NHANES also limited the DI-GM scoring range, possibly underestimating its associations. Fourth, despite comprehensive adjustment for confounders, residual confounding remains inevitable. NHANES lacks critical PSD-related covariates: stroke severity (NIHSS), stroke subtype, onset timing, lesion location, physical/cognitive impairment, antidepressant use, rehabilitation status, social support, and prior mental illness. These unmeasured factors may distort the HEI-2020–PSD correlation, warranting cautious interpretation of our independent association results. Follow-up studies incorporating detailed clinical stroke indicators, psychiatric evaluations, and medication records are needed to replicate our findings. Fifth, the observed inconsistent mediation pattern must be interpreted cautiously due to cross-sectional constraints. This statistical suppression pattern neither confirms nor refutes biological diet–microbiota–depression pathways; future research incorporating metagenomic microbial profiling is necessary to validate potential underlying mechanisms.

Sixth, many participants were excluded for missing data or ineligibility. We confirmed no major demographic differences between included and excluded individuals, and NHANES sampling weights partially offset selection bias, though residual bias cannot be completely eliminated.

## Conclusions

Higher HEI-2020 scores were associated with lower odds of PSD, with a nonlinear dose–response pattern and variation across racial or ethnic groups. Mediation analysis indicated a statistically significant indirect association through DI-GM, an exploratory dietary proxy, but with an inconsistent direction of effects. Given that DI-GM does not directly measure the gut microbiome, these mediation findings should be interpreted with caution and do not provide evidence for a biological gut microbiota-mediated pathway. These findings support a cross-sectional association between dietary quality and lower PSD odds and suggest that HEI-2020 may be useful for risk stratification in observational research. Future prospective studies are needed to determine whether this association reflects a causal relationship that could inform nutritional recommendations. The observed HEI-2020 value was 57.27, which corresponds to the 66th percentile of the sample distribution. This should be interpreted as an exploratory finding for a specific sample. Given the marginal significance of the non-linear test (*P* = 0.049), this observation needs to be independently verified in a prospective cohort before it can be considered for clinical interpretation. Future prospective cohort studies and randomized controlled trials, incorporating metagenomic approaches, are needed to determine whether a biological pathway linking diet, the gut microbiome, and depression exists, and if so, to inform the potential development of nutritional strategies for post-stroke populations.

## Data Availability

Data were obtained from the NHANES database (https://www.cdc.gov/nchs/nhanes/), which is a publicly available and de-identified database managed by the U.S. Centers for Disease Control and Prevention (CDC).

## Abbreviations

CI: Confidence interval
DI-GM: Dietary Index for Gut Microbiota
HEI-2020: Healthy Eating Index-2020
MET: Metabolic Equivalent of Task
NHANES: National Health and Nutrition Examination Survey
OR: Odds ratio
PHQ-9: Patient Health Questionnaire-9
PIR: Poverty income ratio
PSD: Post-stroke depression
